# Retraction Note: Deficiency in intestinal epithelial *Reg4* ameliorates intestinal inflammation and alters the colonic bacterial composition

**DOI:** 10.1038/s41385-020-00367-2

**Published:** 2020-12-16

**Authors:** Yongtao Xiao, Ying Lu, Ying Wang, Weihui Yan, Wei Cai

**Affiliations:** 1 Department of Pediatric Surgery, Xin Hua Hospital, School of Medicine, Shanghai Jiao Tong University, Shanghai, China; 2 Shanghai Institute of Pediatric Research, Shanghai, China; 3 Shanghai Key Laboratory of Pediatric Gastroenterology and Nutrition, Shanghai, China

Correction to: *Mucosal Immunology* **12**, 919–929 (2019); 10.1038/s41385-019-0161-5, published online 5 April 2019

The Editor in Chief has retracted this article^1^ because the validity of data has been called into question and authors were not able to provide full raw data.

Y. Xiao agrees with this retraction. Ying Lu, Ying Wang, Weihui Yan and Wei Cai have not responded to any correspondence from the editor or publisher about this retraction.
